# Myopia disease mouse models: a missense point mutation (S673G) and a protein-truncating mutation of the *Zfp644* mimic human disease phenotype

**DOI:** 10.1186/s13578-019-0280-4

**Published:** 2019-02-21

**Authors:** Katarzyna I. Szczerkowska, Silvia Petrezselyova, Jiri Lindovsky, Marcela Palkova, Jan Dvorak, Peter Makovicky, Mingyan Fang, Chongyi Jiang, Lingyan Chen, Mingming Shi, Xiao Liu, Jianguo Zhang, Agnieszka Kubik-Zahorodna, Bjoern Schuster, Inken M. Beck, Vendula Novosadova, Jan Prochazka, Radislav Sedlacek

**Affiliations:** 1 Laboratory of Transgenic Models of Diseases, Institute of Molecular Genetics CAS, Prumyslova 595, Vestec, 252 50 Prague, Czech Republic; 2 Czech Centre for Phenogenomics, Institute of Molecular Genetics CAS, Prague, Czech Republic; 3 Division of Clinical Immunology, Department of Laboratory Medicine, Karolinska Institutet at Karolinska University Hospital Huddinge, Stockholm, Sweden; 4 BGI-Shenzhen, Shenzhen, 518083 China; 5 China National GeneBank, BGI-Shenzhen, Shenzhen, 518120 China; 6 Animal Research Center, Ulm University, Ulm, Germany

**Keywords:** Myopia, Mouse model, Genetics, Zinc finger 644, Vision, Eye

## Abstract

Zinc finger 644 (Zfp644 in mouse, ZNF644 in human) gene is a transcription factor whose mutation S672G is considered a potential genetic factor of inherited high myopia. ZNF644 interacts with G9a/GLP complex, which functions as a H3K9 methyltransferase to silence transcription. In this study, we generated mouse models to unravel the mechanisms leading to symptoms associated with high myopia. Employing TALEN technology, two mice mutants were generated, either with the disease-carrying mutation (*Zfp644*^*S673G*^) or with a truncated form of *Zfp644* (*Zfp644*^*Δ8*^). Eye morphology and visual functions were analysed in both mutants, revealing a significant difference in a vitreous chamber depth and lens diameter, however the physiological function of retina was preserved as found under the high-myopia conditions. Our findings prove that ZNF644/Zfp644 is involved in the development of high-myopia, indicating that mutations such as, *Zfp644*^*S673G*^ and *Zfp644*^*Δ8*^ are causative for changes connected with the disease. The developed models represent a valuable tool to investigate the molecular basis of myopia pathogenesis and its potential treatment.

**Electronic supplementary material:**

The online version of this article (10.1186/s13578-019-0280-4) contains supplementary material, which is available to authorized users.

## Introduction

Myopia, the most common vision-related disease, is caused by a refractive error [1–3] based on elongation of the axial length of eyes, i.e. by an enlargement of vitreous and anterior chambers and by thinning of lenses but not by retinal dysfunction [1–6]. It is estimated that by the year 2020 myopia will affect 2.5 billion people [3]. The prevalence is increasing over the last few decades [7, 8]. Myopia is dependent on multiple factors such as environmental influences, educational level, diet, or genetics [2, 4, 5, 9–12].

Recently, new genetic factors have been identified to be involved in myopia development [2, 13, 14], calling for new models mimicking the human disease. Among them, *ZNF644* was identified as a potential factor causing inherited myopia in different populations [13, 15–18]. Biological functions and the meaning of mutations found in *ZNF644* are still unclear. So far, twelve mutations have been reported in ZNF644 to be involved in high myopia in humans, a majority of them are localised in exon three [16]. ZNF644 is a protein that binds to G9a (euchromatic histone-lysine *N*-methyltransferase 2, EHMT2) as a part of H3K9 methylation complex together with GLP (euchromatic histone-lysine *N*-methyltransferase 1, EHMT1) [19]. It was shown that ZNF644 and WIZ, another zinc finger protein (Widely-Interspaced Zinc Finger-Containing Protein), interact with G9a and GLP complex respectively. WIZ and ZNF644 are responsible for targeting the G9a/GLP complex to specific DNA loci are also crucial for the regulation of G9a function during transcription [20]. It was also shown that ZNF644 in complex with G9a is present at Active Replication Forks. Knockdown of *ZNF644* in a cell culture results in reduced cell proliferation and higher sensitivity to replication stress as well as an increase of DNA damage in replicating cells [21]. Studies in fish showed that ZNF644 with G9a/GLP complex are responsible for histone methylation critical for gene silencing during neuronal differentiation in retinal neuron differentiation [22].

Examination of myopia in humans is established and employs methods such as optical coherence tomography (OCT), electroretinography (ERG) or ultrasonography (USG) [23–26]. However, investigation of signs of myopia in mouse models is challenging due to the size of eyes [14, 27–29]. It was shown that a 5.4–6.5 μm change in axial length corresponds to one diopter change in refractive error in the eyes of C57BL/6 mice [30]. Change in ocular axial length as small as 100 µm shows high myopia in C57BL/6 mice, thus the selected examination method must be very precise. In this work, we used ophthalmologic examinations known from human medical practice adapted for ophthalmological examinations of large animals [31, 32] and mice [33, 34].

Altogether, we developed and characterized two mouse mutant models of Zpf644. Zfp644^S673G^ that mimics the mutation S672G found in human and Zfp644^Δ8^, which produces a truncated protein product due to a termination codon at position AA673. We experimentally demonstrated that S673G mutation in *Zfp644* is causative of a myopia phenotype and showed that large changes in Zpf644, such as protein truncation, causes a more severe phenotype. All these results point to the important regulatory role of ZNF644 in myopia development. Both Zfp644 mutant models offer new genetic tools for depicting molecular regulatory pathways involved in development of myopia and may shed more light on its potential treatment.

## Materials and methods

### Models generation

All animal models and experiments used in this study were ethically reviewed and performed in accordance with European directive 2010/63/EU and were approved by the Czech Central Commission for Animal Welfare.

TALENs targeting exon 3 of *Zfp644* gene were designed using TAL Effector Nucleotide Targeter 2.0 (https://tale-nt.cac.cornell.edu/) [35, 36] design tool and assembled using Golden Gate cloning method. Left TALENs were designed with 16 RVDs (NN NN NI NG HD NI NI NN HD NG HD NI HD NI NN NG) followed by 16 nt spacer region and right TALENs with 15 RVDs (NN NG NN NN HD HD NN HD NG NG NI NG NN NI NI NI NG). Both TALEN plasmids were used for production of TALEN encoding mRNA as described previously [37].

TALENs mRNA was mixed with a synthetic oligodeoxynucleotide encoding mutated Zfp644 sequence (5′AGGATGCTAAACGGACATTTGGATCATCCAGCCAGAGCGGTAACTTCAGCAAGTTCCACAAGAGACCACATAGAATACAAAAAGCCCGG 3′). Targeting constructs were microinjected into male pronuclei of zygotes from C57BL/6N mice. Two lines of transgenic animals were obtained: 1/Zfp644^S673G^ with desired mutation and 2/Zfp644^Δ8^ allele with the frame shift mutation leading to a STOP codon. The animals were further maintained on C57BL/6N background. For genotyping DNA extracted from tails of 3 weeks old C57BL/6N mice using the Quick Extract DNA Extraction Solution 1.0 Kit (Illumina, USA) was used as template for PCR with following primers (forward primer for Zfp644^Δ8^ 5′-ATCAAGCTCACAGTCAAGTAATTTT-3′; forward primer for Zfp644^S673G^: 5′-TCAGCAAGTTCCACAAGAGACC-3′; reverse primer for both alleles: 5′-TTGTTGGTCAGTGCTGCTCTTAAC-3′).

### Histology

The mice were euthanized by cervical dislocation. Eyes were sampled immediately, inserted into labelled histological cartridges, fixed in Davidson’s solution for 24 h, put into 70% ethanol solution to process using an automated tissue processor (Leica ASP 6025, Leica Microsystems, Germany), and embedded in paraffin blocks using a Leica EG 1150H paraffin embedding station (Leica Microsystems, Germany). Sections of 3-5 μm were cut using a microtome (Leica RM2255, Leica Microsystems, Germany) on standard glass slides (Waldemar Knittel, GmbH, Germany). Eye samples were cut under the stereomicroscope view control and only medial cuts with optic nerve were selected for morphometry. Sections were stained with haematoxylin–eosin and mounted using Ventana Symphony H&E Slide Stainer (Ventana Medical Systems, Inc., USA). The second set of samples were cut and fixed on salinized slides (Thermo Scientific, USA) and used for immunohistochemical procedures.

### RNA in situ hybridization

Digoxygenin-labeled RNA probes (DIG RNA labeling Kit, Roche, Germany) for In Situ Hybridization (ISH) were generated by in vitro transcription from plasmid contained fragment of murine Z*fp644*. Procedure were carried out according to standard protocol [38] on E9.5 after fixation in 4% PFA in whole-mount and 2 µm paraffin section for E12.5 and E14.5. Solutions used: Blocking solution (Roche, Germany), DIG-antibody (Roche, Germany), FastRed (SigmaAldrich, USA), DAPI mounting media (Molecular Probes, USA). For fluorescence and bright-field imaging, Zeiss ImagerZ2 (Zeiss, Germany) was used, for whole-mount Zeiss Apotome (Zeiss, Germany) was used.

### Morphometric analysis

Samples were evaluated using a light-microscopic images obtained using a Carl Zeiss Axio Scope A1 (Zeiss, Germany) and the Axio Scan Z1 slide scanner (Zeiss, Germany).

### qPCR analysis

RNA was isolated and used as a template for reverse transcription into cDNA with M-MLV Reverse Transcriptase (Promega, USA). Quantative PCR (qPCR) reactions were performed using the TATAA SYBR^®^ GrandMaster^®^ Mix (TATAA Biocenter Sweden) in Cycler LightCycler^®^ 480 Instrument II (Roche, Germany). Expression levels of the genes of interest were normalized to levels of *Hprt1* and *Ppia* and are presented as levels relative to wild type control. Primers were designed and ordered from (TATAA Biocenter), sequences are available upon request. All experiments were performed independently in triplicates on 3 different specimens (n ≥ 3) per group.

### Optical coherence tomography (OCT)

Both retinal fundi of 15 Zfp644^S673G^ and 21 Zfp644^Δ8^ homozygous mice with 25 respective controls were examined. All animals were 16 weeks old. All mice were anaesthetized with 20% Zoletil–tiletamin 0.03 g/kg and zolazepam 0.03 mg/g (Virbac, France). Pupils of eyes were dilated using eye-drops Atropin-POS 0.5% (Ursapharm, Czech Republic). To prevent the corneal dehydration, the aqueous eye gel Vidisic 1 × 10 mg (Dr. Gerhard Mann Pharma, Germany) was applied on the eyes and subsequently, PMMA contact lens (Cantor&Nissel, UK) were placed on the eyes. For the image acquisition of the retinal fundus, optical coherence tomography (OCT Spectralis™Plus, HRA Spectralis System Heidelberg Engineering GmbH, Heidelberg, Germany) with a 30° lens was used. Mice were placed on a platform fixed in front of the OCT camera and the eye horizontally directed toward the camera; the fundus was focused and cross-sectional images were taken. The segmentation of retinal layers, retinal thickness, optic disc position and blood vessels pattern were analyzed from the high-resolution cross-sectional images using HRA/Spectralis Calculation Data Manager. The average of retinal thickness was calculated from values measured in the medial cross-section in the distance of 2 mm to the nasal and temporal side of fundus from the optic disc (Additional file 1: Figure S2D).

### Electroretinography (ERG)

ERG was performed under general anaesthesia as described above. Animals were kept on a heating pad at 37 °C with eyes protected against drying by applying transparent eye gel (Vidisic, Bausch + Lomb, Czech Republic). All measurements were done on the right eye 10 min after application of 0.5% solution of atropin (Ursapharm, Czech Republic). Animals were adapted to darkness for 12 h (over night) prior to the experiment. When the scotopic part of the stimulation protocol terminated, the mice were exposed to white background light (25 cd/m^2^) at least for 2 min before start of the photopic stimulation protocol. The ERG stimulation and recording setup (RETI-port for animal, Roland Consult, Germany) allowed single-flash stimuli to be applied to the whole retina by ganzfeld equipped with LED diods and Xenon lamp, luminances were logarithmically distributed between 0.003 and 100 cd s/m^2^. A golden ring (3 mm in diameter) was placed on the cornea as the active electrode, a golden wire inserted in the animal’s mouth served as the reference electrode. Each stimulus was repeated 7–10 times and an averaged signal was saved. The signal was band-pass filtered between 1 and 300 Hz and digitized with 2 kHz sampling frequency. The scotopic and photopic responses were inspected off-line using a custom-made script in Matlab (MathWorks), a-wave parameters were measured in the original recording whereas b-wave parameters were quantified after removal of the oscillatory potentials from the recordings by low-pass filtering with 80 Hz cut-off frequency.

### Ultrasound imaging (USG)

Ophthalmologic ultrasound measurements were performed on 12–14 weeks old mice. Ultrasound imaging was acquired by a Vevo 2100 Imaging System (FUJIFILM VisualSonics, Inc., Toronto, ON, Canada) equipped with a MS-550S transducer operating at a center frequency of 44 MHz. The MS550S has axial resolution of 40 μm at its focal depth and allows revealing both the anterior and posterior structures of the mouse eye (Fig. 3a). For ophthalmic ultrasound imaging, mice were anesthetized with 1–2.5% isoflurane in oxygen (1 L/min) and body temperature was maintained at 37 °C. Care was taken to place the subjects in similar postures to ensure similar orientation. Sterile hypoallergenic ultrasound gel without any air bubbles was applied between the eye and the transducer and subsequently eyes were imaged. The eye structures were measured by manually delineating margins using Vevo^®^LAB V1.7.0. Software. The software then calculated the corresponding length of each eye.

### Western blotting

Organs were collected from adult male mice and homogenized using beads in Tissue Lyzer II (Qiagen, Germany) in NETN400 Lysis buffer (0.5% NP-40, 50 mM Tris–HCl pH 8.0, 2 mM EDTA, 400 mM NaCl, 10 mM NaF, 50 mM β-glycerophosphate) containing protease inhibitors. Protein lysates were centrifuged at 4 °C at 3000 rpm for 5 min. The supernatant was carefully removed and pellets containing large plasma membrane pieces, DNA and nucleoli were diluted with NETN0 Lysis buffer (no containing salts) to 100 mM NaCl. Protein concentration of nuclear fractions were determined by Pierce BCA Protein Assay Kit (Thermo Scientific, USA). Samples were loaded on 8% SDS-PAGE gel and transferred onto nitrocellulose membrane (GE Healthcare Life Science, Germany). Membranes were blocked for at least 1 h in 5% milk in TBS-T before incubating overnight at 4 °C with the appropriate primary antibody. Antibodies used were anti-ZNF644 and anti-GAPDH (G9545, Sigma-Aldrich, USA). The anti-ZNF644 (raised against N-terminus AA50-602) was kindly provided by Xiaochun Yu. The following day, membranes were washed with TBS-T, incubated with appropriate secondary antibody (Sigma-Aldrich, USA) at room temperature for 1 h and then washed again with TBS-T. Membranes were developed using Pierce™ ECL Western Blotting substrate (Thermo Scientific™, USA) and images captured using a ChemiDoc™ detection system (Bio-Rad).

### Statistical analyses

Statistical analysis from ultrasonography examination was performed using GraphPad Prism software version 7.0 (GraphPad, USA); data was analyzed with one-way ANOVA. Data from OTC examination was performed in R software version 3.3 (R Core Team, Austria) using linear mixed model. Data from qPCR was analyzed using Genex 6.1 (MultiD, Sweden); qPCR statistic, analysis and graphs was performed in R software version 3.3 (R Core Team, > Austria).

## Results

### *Zfp644* is expressed in developing and adult mouse eye

**Fig. 1:**
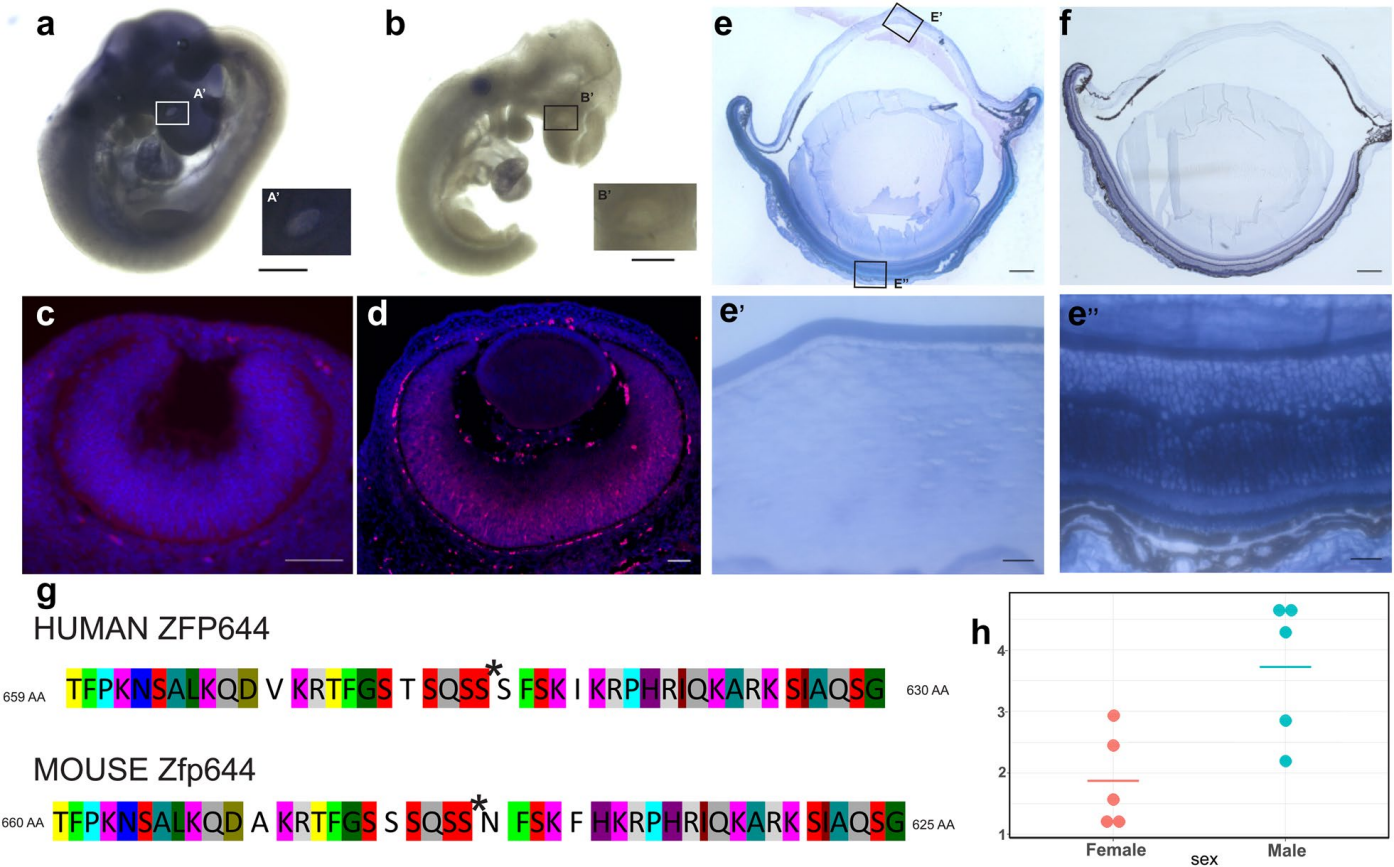
Zfp644 is expressed in mouse embryonic and adult eye. **a** Expression of *Zfp644* gene in embryonic eye at E9.5,; scale bar, 500 µm. (A’) Focus on eye **b** Negative control; E9.5; scale bar, 500 µm. (B’) Focus on eye **c** Expression of *Zfp644* gene in eye of E 12.5; scale bar, 50 µm. **d** Expression of *Zfp644* gene in eye of E 14.5; scale bar, 50 µm. **e** Expression of Zfp644 in adult eye; scale bar, 500 µm. **e**’ Expression of Zfp644 in adult lens; scale bar, 100 µm. **e**” Expression of Zfp644 in adult retina scale bar, 100 µm. **f** Negative control; adult eye; **g** Protein sequence alignment of human ZNF644 and mouse Zfp644 showing position of the single point mutation at the amino acid at position 673 (asterisk); **h** Expression of *Zfp644* gene in eyes of WT animals

To investigate the expression pattern of Zfp644 in embryonic development, we performed whole-mount ISH of mouse embryos at E9.5 (early development of mouse eye) and fluorescence ISH of mouse embryos at E12.5 (ocular tissues differentiation) and E14.5 (corneal development). The embryonic eyes exhibited a strong hybridization signal (Fig. 1a, c, d). We also noticed strong expression in the brain and in a cervical part of a developing nervous system but not in proximal nor distal parts of spinal cord (Fig. 1a). This study was performed also on adult eyes, showing the signal in the retina and lens (Fig. 1e, e’’). The level of Zfp644 expression in adult eyes was quantified in samples isolated from male and female eyes by qRT-PCR, which showed very interesting gender dependent pattern with a higher expression level in male eyes (Fig. 1h).

### Generation of Zfp644 mutant models

**Fig. 2:**
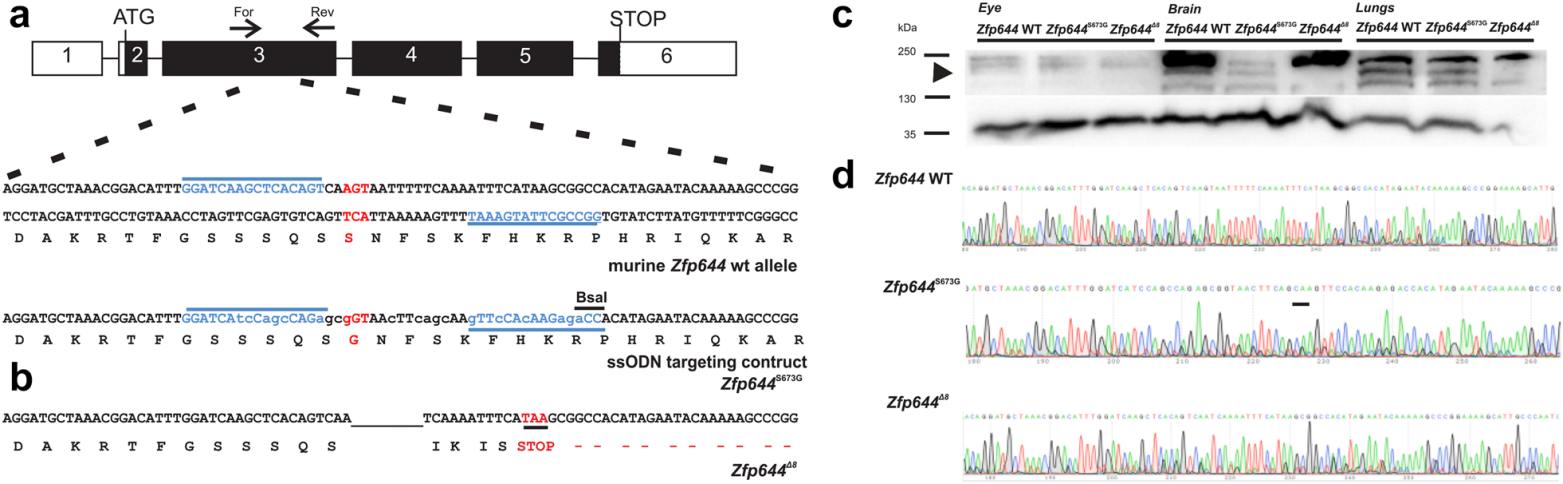
Generation of *Zfp644* mutant mice. **a** Schematic representation of murine Z*fp644* gene with depiction of the targeted DNA sequence. Position of critical AGT nucleotides (red) in the exon 3 of murine Z*fp64*4. TALEN binding sites are marked with blue underline, position of primers for PCR screening (For, Rev) are denoted. **b** Sequence of Zfp644^Δ8^ with highlighted deletion of 8 nucleotides (black underline) and a STOP codon (red letters) is shown. **c** In every examined tissue of Zfp644^Δ8^animals no detectable protein expression of Zfp644 can be found, while the samples of Zfp644^S673G^ and control animals show protein expression in every examined tissue. Black arrow indicates correct size of Zfp644 protein (145 kDa). Expression of GAPDH protein was used as a reference protein. **d** Both mutations were confirmed by sequencing in both G1; and cDNA of adult mice eyes, brain and lungs of transgenic and control animals

Due to a high similarity between murine and human nucleotide sequences (Fig. 1g), the first model mimics S672G mutation in *ZNF644* gene (S673G in mouse), described previously in a patient with the mutation suggested to be causative of inherited high myopia in humans (Fig. 2a). To generate the model, TALEN nucleases (TALENs) were used to specifically targeted the region in Z*fp644* gene in combination with single-stranded oligodeoxynucleotide (ssODN) carrying the desired mutation (Fig. 2a). In addition to the insertion of ssODN with the point mutation, we also obtained a mutant with deletion of eight amino acids resulting in formation of STOP codon at position AA673, which leads to a truncation of *Zfp644* (Fig. 2b). To confirm a formation of truncated form of the Zfp644 protein we performed a Western Blot analysis with anti-ZNF644 antibody (Fig. 2c). The expected size of Zfp644 protein is 145 kDa, as showed in Fig. 2c, a band this size was missing in all examined organs from Zfp644^Δ8^ animals. Both mutations were confirmed by sequencing also in G1 and cDNA of adult mice (Fig. 2d).

### Mutations in *Zfp644* lead to myopia caused by enlargement of the optical axis

**Fig. 3:**
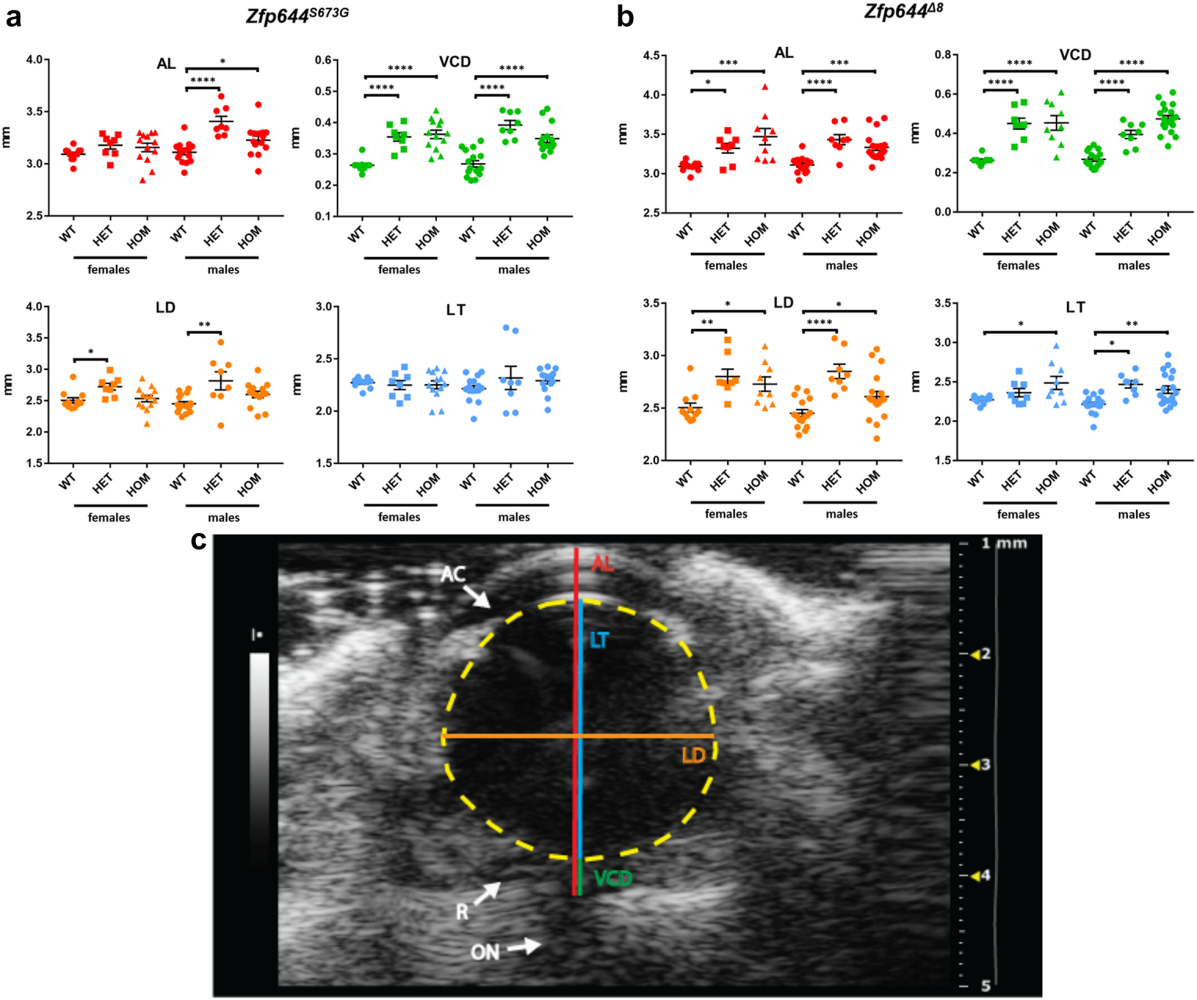
Analysis of ocular parameters measurement with an ultrasound. **a**, **b** Four different ocular length parameters were measured: AL (axial length; in red), VCD (vitreous chamber depth; in green), LD (lens diameter; in orange) and LT (lens thickness; in blue). Analysis of ocular length parameters between groups in Zfp644^Δ8^ (**a**) and Zfp644^S673G^ (**b**); Most prominent differences were detected in axial length (ANOVA, p < 0.0392 − 0.0001) and vitreous chamber diameter (ANOVA, p < 0.0001); **c** Typical ultrasound image of the mouse eye; Four ocular length parameters are shown on a typical ultrasound image of an eye: AL (axial length; in red), VCD (vitreous chamber depth; in green), LD (lens diameter; in orange) and LT (lens thickness; in blue). The lens border is depicted by yellow dashed line. White arrows indicate anterior chamber (AC) and retina (R)

Myopia is a vision-related disease caused by the elongation of the axial length of an eye. To investigate the impact of *Zfp644* mutations on multiple ocular parameters in vitro we employed an ultrasound imaging technique. High-frequency ultrasound imaging [39–42] was used to image posterior structures of the eye, and in particular, the retina and optical nerve was performed with the MS-550S transducer using an imaging depth of 6 to 7 mm with axial resolution of 40 μm, thus providing a satisfied visibility for posterior eye features (Fig. 3c and Additional file 2: Figure S1). Ultrasound images of eyes with depicted ocular parameters measured in this study i.e. axial length (AL), vitreous chamber depth (VCD), lens diameter (LD) and lens thickness (LT) are shown in Fig. 3c. Ophthalmic ultrasound examinations were performed on homozygous and heterozygous Zfp644^S673G^ mice (Fig. 3a), Zfp644^Δ8^ mice (Fig. 3b), and the corresponding control (WT) mice at the age of 12–14 weeks.

In Zfp644^S673G^ animals (Fig. 3a and Additional file 3: Table S1), a significant difference in optical axis enlargement was observed only in males and, more strikingly, heterozygote constitution showed higher penetrance of a phenotype than homozygote males suggesting weak dominant negative effect of S673G mutation with gender specific bias. Nevertheless, despite no significant difference was found in total axial length in females, a vitreous chamber depth was significantly different in both, the heterozygous and homozygous animals, without gender influence. Similar to Zfp644^Δ*8*^ animals, changes in lens diameter were most significant in heterozygous animals.

In Zfp644^Δ8^ animals, alteration of the axial length is caused by morphological changes in vitreous chamber depth as well as in lens thickness and diameter (Fig. 3b and Additional file 4: Table S2). Our analysis showed that every component of the optical axis is affected by the mutation. Differences in individual ocular parameters result in enlargement of the optical axis, which results in a/the high myopia phenotype. A significant difference in all measured parameters between control and mutated mice was observed besides only one exception, the lens thickness in heterozygous females from Zfp644^Δ8^ mice, which showed no significant difference. However, a tendency towards a high median value in heterozygous animals was evident.

In summary the protein-truncated mutation, Zfp644^Δ*8*^, caused a more severe phenotype then the point mutation, which is visible in both sexes and affects also heterozygotes. In contrast, S673G is less penetrant and more pronounce in males, however mild changes leading to optical axis enlargement were observed also in females. Interestingly, in the case of S673G mutation, the eye morphology was affected more in heterozygous constitution in multiple parameters. Altogether, these results suggest dominant negative behavior of the mutated form of *Zfp644* and closely mimics the situation reported in human patients [13].

### Morphology and function of the retina remains unchanged in the mutant models

**Fig. 4:**
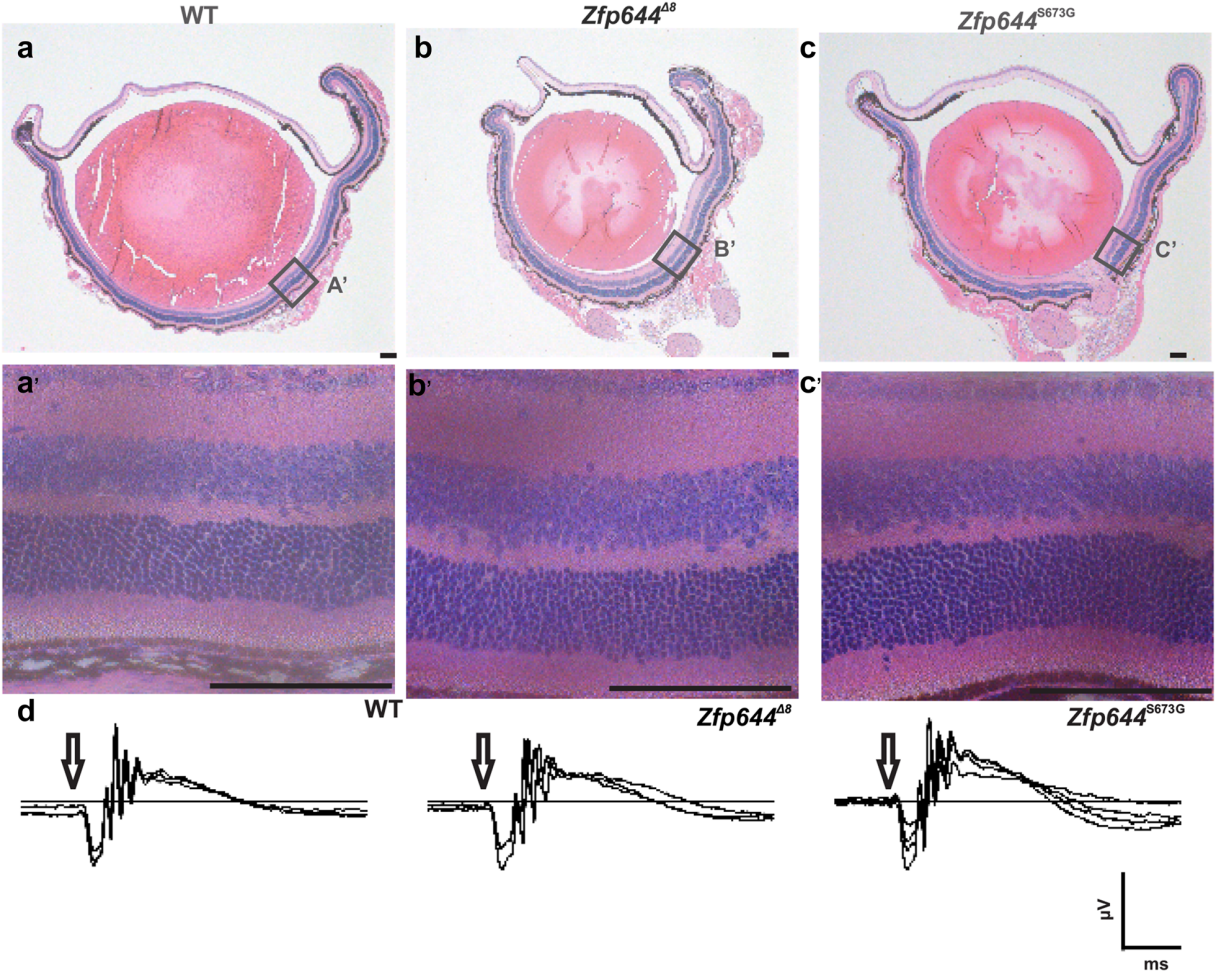
Function and morphology of retina remain unchanged in transgenic animals. **a**–**c** Histological examination of retina, was done on 5 weeks old and 16 weeks old animals and compared between WT and transgenic animals. Representative image of eye sections of 5 weeks old animals are presented (**a**–**c**); scale bar, 100 µm. Zoom in on retina; scale bar, 100 µm (**a**’–**c**’); Positions of zooming are denoted with black square (**d**). Function of retina was examined by ERG measurement in 21 weeks old animals and shows no difference between WT and transgenic animals. Example responses to single white flash (3.0 cd s/m^2^) are shown, arrows denote the time of stimulation. Scale bar 50 ms and 200 µV

As showed previously in the fish model, *ZNF644* exhibited a severe impact on retinal function and morphology [20]. To confirm these findings, we examined morphology and function of retina in mouse *Zfp644* mutants. Both eyes of Zfp644^S673*G*^ (n = 15) and Zfp644^Δ8^ (n = 21) homozygous mutants at an age of 16 weeks were examined with OCT and compared with 25 age-matched wildtype controls. Retinal layer segmentation, optic disc position and blood vessel patterning was assessed. None of these parameters showed significant differences (Additional file 1: Figure S2A–C’). Histopathological examination of retinal structure also showed no significant difference (Fig. 4a–c’). However, in vivo imaging by OCT across the optic disc showed a significant difference in retinal thickness between control and transgenic animals (Additional file 1: Figure S2D–F). The distribution of retinal thickness in transgenic animals showed a thinner retina than in control animals. However, examination of retinal thickness on morphology sections, of different sections of retina, did not show a significant difference, but a tendency in Zfp644^Δ8^ animals remain the same as difference found in OCT analysis (Additional file 1: Figure S2G). Overall, retinal thickness analysis showed a tendency to thin between control and transgenic animals. In order to provide functional validation of morphological findings we conducted electrophysiological measurements to assess the response of photoreceptors and other neurons to light stimulation by ERG. Amplitudes and implicit times of scotopic and photopic wave a (response of photoreceptors) and wave b (response of bipolar cells) respectively, were measured, however no abnormalities were found in the mutants (Fig. 4d and Additional file 5: Figure S4A–B). In addition, quantification of cell numbers in individual retina layers (ganglion cells, outer layer cells and inner layer cells) did not reveal any significant difference (Additional file 6: Figure S3A–D). These results suggest very low or no impact of *Zpf644* on the function and morphology of retina.

## Discussion

Only a limited number of genetic mouse models of myopia have been generated so far. *Nob*^*null*^ mice, first described as a model for human night blindness [43], were found to be susceptible for a form of deprivation myopia [44–46]. Other models such as Aplp2-deficient mice [47], *P3h2*^*n/n*^ [48] or lumican transgenic mouse model [14] mimic the patient’s phenotype only partially. Although, myopia can be studied in animals with disease induced during mouse life (e.g. by googles, no developmental, molecular nor genetic factor can be investigated [49, 50]).

In this study, we generated two novel mouse models Zfp644^S673G^ and Zfp644^Δ8^ that conclusively mimic human inherited high myopia. We showed that both mouse models carry signs of a myopia and the phenotype is stronger in mice with truncated Zfp644 (Zfp644^Δ8^) than in point mutation. Moreover, we also showed that measured ocular parameters are significantly different in heterozygote animals when compared to WT animals, which corresponds with genetic conditions reported previously [13, 17].

No *Zfp644*-deficient mouse model has been generated so far, and only a fish model deficient for *ZNF644* was previously described [22]. To investigate the molecular mechanism of *ZNF644*, Olsen et al. [22] prepared two different morphant models (MO) of *znf644*, based on two *znf644* isoforms (a and b) whose phenotype was severe and included changes in developing retina, midbrain, and eye size. Both znf644a-MO and znf644b-MO showed signs of microphthalmia and disrupted midbrain morphology. Nevertheless, we could not reveal significant differences in retinal morphology, cell number in the retinal layers or in the electrophysiological responses of the retina, in any of *Zfp644* mutant mouse models.

An interesting feature of mouse Zfp644^S673G^ mutation is not only recapitulation of human disease but there is also stronger phenotype manifestation in males. The mechanism of this phenomenon is not easy to explain but it could be a result of a different basal level of *Zfp644* expression in male and female eyes. The candidacy of *Zfp644* in steroid hormones signaling is also supported by the work of Davis [51], in which upregulated *Zfp644* expression was observed in 8 week-old ovariectomized mice following treatment with estradiol, a steroidal sex hormone.

This could also suggest potential role of steroid hormones signaling in regulation of ZNF644, although published human case reports suggested no gender specificity in any of the mutation variants of *ZNF644* [13, 15–17].

Taking in account that myopia is not a retina related disease, the mouse model provides better opportunities to study the molecular role of *ZNF644* in human patients with inherited high myopia, then lower vertebrate model. Therefore, genetically modified mouse models presented in this study, are advantageous mammalian models to study genetic regulations causing inherited high myopia in humans and might serve as development base for testing of potential novel therapeutic strategies.

## Additional files

**Additional file 1: Figure S2.** Typical view of the fundus with the optic disc and blood vessels in the WT **(A),** Zfp644^S673G^ **(B)** and Zfp644^Δ8^ **(C)**. The white spot is a reflected light. A typical view of the retinal cross-sections thru the optic disc, respectively **(A‘-C‘)**. ILM – internal limiting membrane, BM – Bruch´s membrane. Scale bar; 200 µm. **(D)** Retinal thickness profile; position of the optic nerve is marked with a red arrow, green arrows indicate a distance in temporal and nasal retina were the retinal thickness values were collected. Retinal thickness was measured as an average of five measurements between green arrows on both temporal and nasal parts of fundus, starting from 0,5 µm from the middle of the optic disc, thru the nasal or temporal retina in 1,5 µm distance. **(E)** Thickness of the retina measured on morphological sections is showed in a box plot. **(F)** Linear distribution of the retinal thickness is showed in a plot. **(G)** Statistical analyses of retinal thickness distribution are showed in a box plot.

**Additional file 2: Figure S1.** Representative USG image of mice eyes. Both males and females eyes of every examined group are presented.

**Additional file 3: Table S1.** Summary of the results of ophthalmologic ultrasound measurements on WT, HET, and HOM Zfp644^S673G^ ^8^ eyes. For each sex, medians, first and third quartile and p-values were calculated by one-way ANOVA analysis comparing WT, HET and HOM eyes.

**Additional file 4: Table S2.** Summary of the results of ophthalmologic ultrasound measurements on WT, HET, and HOM Zfp644^Δ^ eyes. For each sex, medians, first and third quartile and p-values were calculated by one-way ANOVA analysis comparing WT, HET and HOM eyes.

**Additional file 5: Figure S4.** Electroretinography. Whole-field electroretinography was recorded in animals adapted to darkness (scotopic condition) and the same animals adapted to light background (photopic condition). A) Example responses obtained in three different animals, Zfp644 WT, left column, Zfp644 S673G, middle column, and Zfp644 Δ8, right column, respectively. The time of light flash is marked with vertical dotted lines. Responses represent an averaged signal of seven to ten successive stimulations. B) Amplitude of the responses, top row, and their implicit time, bottom row, summerized for all animals and all flash luminances used. Circles represent result obtained in individual animals, lines show the mean values of each genotype, n = 4 (Zfp644 WT), n = 3 (Zfp644 S673G), n = 5 (Zfp644 Δ8). Neither the response of photoreceptors, as represented by the a-wave parameters, left column, nor the response of ON type of bipolar cells represented by b-wave, middle and right column, was significantly different between WT and mutated animals.

**Additional file 6: Figure S3.** Evaluations of retina cell numbers. Cells were counted in 200 µm. Four measurements are showed here: **(A)** cells in outer layer; **(B)** cells in inner layer; **(C)** ganglion cells; **(D)** and a total cells number. No significant differences were found.
