# Supplementary figures and images for: Myopia disease mouse models: a missense point mutation (S673G) and a protein-truncating mutation of the Zfp644 mimic human disease phenotype
Source: Cell Biosci. 2019 Feb 21;9:21. doi: 10.1186/s13578-019-0280-4 (PMC6385473; doi:10.1186/s13578-019-0280-4)

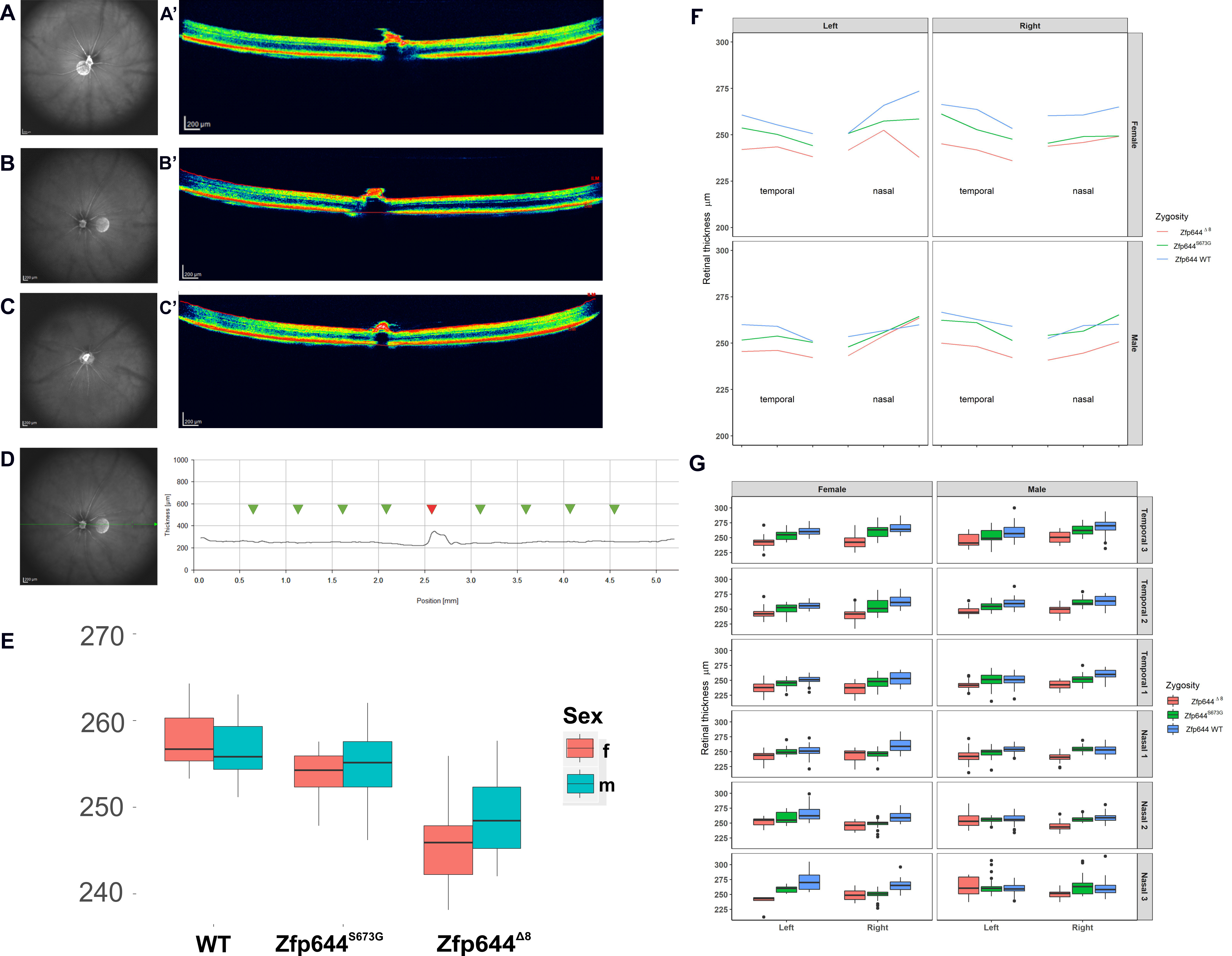

Supplement: Supplementary file 1 — Additional file 1: Figure S2. Typical view of the fundus with the optic disc and blood vessels in the WT (A), Zfp644S673G (B) and Zfp644Δ8 (C). The white spot is a reflected light. A typical view of the retinal cross-sections thru the optic disc, respectively (A‘-C‘). ILM – internal limiting membrane, BM – Bruch´s membrane. Scale bar; 200 µm. (D) Retinal thickness profile; position of the optic nerve is marked with a red arrow, green arrows indicate a distance in temporal and nasal retina were the retinal thickness values were collected. Retinal thickness was measured as an average of five measurements between green arrows on both temporal and nasal parts of fundus, starting from 0,5 µm from the middle of the optic disc, thru the nasal or temporal retina in 1,5 µm distance. (E) Thickness of the retina measured on morphological sections is showed in a box plot. (F) Linear distribution of the retinal thickness is showed in a plot. (G) Statistical analyses of retinal thickness distribution are showed in a box plot. [file 13578_2019_280_MOESM1_ESM.png]

# Female

# Male

**Zfp644 WT**

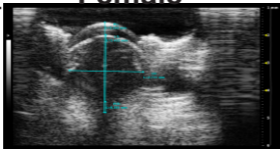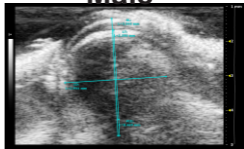

hom

het

hom

het

**Zfp644<sup>S673G</sup>**

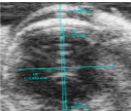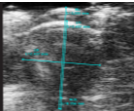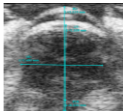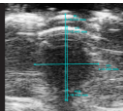

**Zfp644<sup>Δ8</sup>**

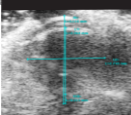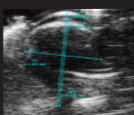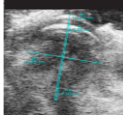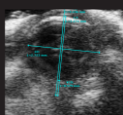

Supplement: Supplementary file 2 — Additional file 2: Figure S1. Representative USG image of mice eyes. Both males and females eyes of every examined group are presented. [file 13578_2019_280_MOESM2_ESM.pdf]

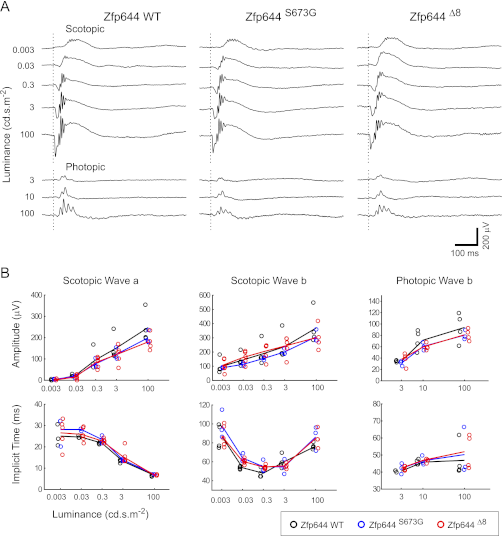

Supplement: Supplementary file 5 — Additional file 5: Figure S4. Electroretinography. Whole-field electroretinography was recorded in animals adapted to darkness (scotopic condition) and the same animals adapted to light background (photopic condition). A) Example responses obtained in three different animals, Zfp644 WT, left column, Zfp644 S673G, middle column, and Zfp644 Δ8, right column, respectively. The time of light flash is marked with vertical dotted lines. Responses represent an averaged signal of seven to ten successive stimulations. B) Amplitude of the responses, top row, and their implicit time, bottom row, summerized for all animals and all flash luminances used. Circles represent result obtained in individual animals, lines show the mean values of each genotype, n = 4 (Zfp644 WT), n = 3 (Zfp644 S673G), n = 5 (Zfp644 Δ8). Neither the response of photoreceptors, as represented by the a-wave parameters, left column, nor the response of ON type of bipolar cells represented by b-wave, middle and right column, was significantly different between WT and mutated animals. [file 13578_2019_280_MOESM5_ESM.png]

**A****Outer layer**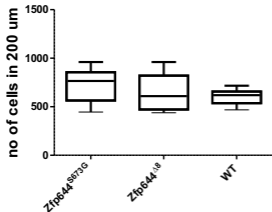**B****Inner layer**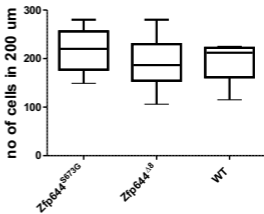**C****Ganglion cells**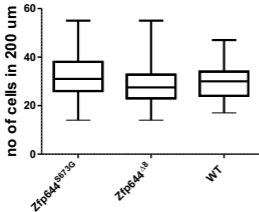**D****Total cells number**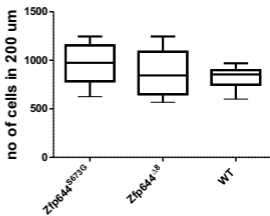

Supplement: Supplementary file 6 — Additional file 6: Figure S3. Evaluations of retina cell numbers. Cells were counted in 200 µm. Four measurements are showed here: (A) cells in outer layer; (B) cells in inner layer; (C) ganglion cells; (D) and a total cells number. No significant differences were found. [file 13578_2019_280_MOESM6_ESM.pdf]
