# Supplementary material for: Myopia disease mouse models: a missense point mutation (S673G) and a protein-truncating mutation of the Zfp644 mimic human disease phenotype
Source: Cell Biosci. 2019 Feb 21;9:21. doi: 10.1186/s13578-019-0280-4 (PMC6385473; doi:10.1186/s13578-019-0280-4)
Supplement: Supplementary file 3 — Additional file 3: Table S1. Summary of the results of ophthalmologic ultrasound measurements on WT, HET, and HOM Zfp644S673G 8 eyes. For each sex, medians, first and third quartile and p-values were calculated by one-way ANOVA analysis comparing WT, HET and HOM eyes. [file 13578_2019_280_MOESM3_ESM.docx]

| **Table S1: Summary of the results of ophthalmologic ultrasound measurements on WT, HET and HOM *Zfp644^S673G^* eyes.** For each sex, medians, first and third quartile and p-values were calculated by one-way ANOVA analysis comparing WT, HET and KI eyes. | | | | | | | | | |
| --- | --- | --- | --- | --- | --- | --- | --- | --- | --- |
| Parameter | female | | | | male | | | female | male |
|  | WT | | HET | KI | WT | HET | KI |  |  |
|  | n=11 | | n=8 | n=13 | n=16 | n=8 | n=14 | p-value | p-value |
|  | median  [25%, 75%] | | | | | | |  |  |
| LT (mm) | 2.285  [2.25, 2.295] | 2.27  [2.136, 2.351] | | 2.259  [2.189, 2.381] | 2.215  [2.194, 2.285] | 2.272  [1.978, 2.652] | 2.329  [2.251, 2.368] | WT vs. HET  ns  WT vs. KI  ns | WT vs. HET  ns  WT vs. KI  ns |
| LD (mm) | 2.465  [2.407, 2.583] | 2.724  [2.577, 2.818] | | 2.567  [2.423, 2.667] | 2.426  [2.334, 2.583] | 2.837  [2.578, 3.09] | 2.61  [2.515, 2.711] | WT vs. HET  p=0.0142  WT vs. KI  ns | WT vs. HET  p=0.002  WT vs. KI  ns |
| AL (mm) | 3.096  [3.047, 3.125] | 3.213  [3.102, 3.269] | | 3.208  [3.056, 3.286] | 3.129  [3.036, 3.166] | 3.35  [3.289, 3.526] | 3.237  [3.141, 3.3] | WT vs. HET  ns  WT vs. KI  ns | WT vs. HET  p=0.0001  WT vs. KI  p=0.0321 |
| VCD (mm) | 0.264  [0.254, 0.264] | 0.358  [0.322, 0.387] | | 0.366  [0.323, 0.405] | 0.2541  [0.237, 0.308] | 0.387  [0.351, 0.436] | 0.332  [0.321, 0.368] | WT vs. HET  p=0.0001  WT vs. KI  p=0.0001 | WT vs. HET  p=0.0001  WT vs. KI  p=0.0001 |
| LT, lens thickness; LD, lens diameter; AL, axial length; VCD, vitreous chamber depth; ns, non-significant | | | | | | | | | |
