# Supplementary material for: Myopia disease mouse models: a missense point mutation (S673G) and a protein-truncating mutation of the Zfp644 mimic human disease phenotype
Source: Cell Biosci. 2019 Feb 21;9:21. doi: 10.1186/s13578-019-0280-4 (PMC6385473; doi:10.1186/s13578-019-0280-4)
Supplement: Supplementary file 4 — Additional file 4: Table S2. Summary of the results of ophthalmologic ultrasound measurements on WT, HET, and HOM Zfp644Δ eyes. For each sex, medians, first and third quartile and p-values were calculated by one-way ANOVA analysis comparing WT, HET and HOM eyes. [file 13578_2019_280_MOESM4_ESM.docx]

| **Table S2: Summary of the results of ophthalmologic ultrasound measurements on WT, HET and HOM**  Zfp644^Δ8^ **eyes.** For each sex, medians, first and third quartile and p-values were calculated by one-way ANOVA analysis comparing WT, HET and KO eyes. | | | | | | | | | |
| --- | --- | --- | --- | --- | --- | --- | --- | --- | --- |
| Parameter | female | | | | male | | | female | male |
|  | WT | | HET | KO | WT | HET | KO |  |  |
|  | n=11 | | n=8 | n=9 | n=16 | n=8 | n=19 | p-value | p-value |
|  | median  [25%, 75%] | | | | | | |  |  |
| LT (mm) | 2.285  [2.25, 2.295] | 2.316  [2.23, 2.473] | | 2.387  [2.283, 2.697] | 2.215  [2.194, 2.285] | 2.502  [2.351, 2.561] | 2.329  [2.234, 2.59] | WT vs. HET  ns  WT vs. KO  p=0.0149 | WT vs. HET  p=0.002  WT vs. KO  p=0.0037 |
| LD (mm) | 2.465  [2.407, 2.583] | 2.745  [2.708, 2.968] | | 2.642  [2.545, 2.908] | 2.426  [2.334, 2.583] | 2.797  [2.705, 3.054] | 2.58  [2.535, 2.655] | WT vs. HET  p=0.003  WT vs. KO  p=0.0192 | WT vs. HET  p=0.0001  WT vs. KO  p=0.0309 |
| AL (mm) | 3.096  [3.047, 3.125] | 3.355  [3.149, 3.428] | | 3.454  [3.181, 3.651] | 3.129  [3.036, 3.166] | 3.43  [3.344, 3.607] | 3.276  [3.229, 3.364] | WT vs. HET  p=0.0392  WT vs. KO  p=0.0007 | WT vs. HET  p=0.0001  WT vs. KO  p=0.0002 |
| VCD (mm) | 0.264  [0.254, 0.264] | 0.443  [0.386, 0.527] | | 0.433  [0.362, 0.557] | 0.254  [0.237, 0.308] | 0.401  [0.332, 0.444] | 0.472  [0.425, 0.528] | WT vs. HET  p=0.0001  WT vs. KO  p=0.0001 | WT vs. HET  p=0.0001  WT vs. KO  p=0.0001 |
| LT, lens thickness; LD, lens diameter; AL, axial length; VCD, vitreous chamber depth; ns, non-significant | | | | | | | | | |
